# A detailed in silico analysis of secondary metabolite biosynthesis clusters in the genome of the broad host range plant pathogenic fungus *Sclerotinia sclerotiorum*

**DOI:** 10.1186/s12864-019-6424-4

**Published:** 2020-01-02

**Authors:** Carolyn Graham-Taylor, Lars G. Kamphuis, Mark C. Derbyshire

**Affiliations:** Centre for Crop and Disease Management, School of Molecular and Life Sciences, Curtin University, Bentley, Perth, Western Australia Australia

**Keywords:** Subtelomere, Pseudogenisation, Gene loss, Phytotoxin, Necrotroph, Biosynthetic gene cluster, Genomic comparison, Botcinic acid, Melanin

## Abstract

**Background:**

The broad host range pathogen *Sclerotinia sclerotiorum* infects over 400 plant species and causes substantial yield losses in crops worldwide. Secondary metabolites are known to play important roles in the virulence of plant pathogens, but little is known about the secondary metabolite repertoire of *S. sclerotiorum*. In this study, we predicted secondary metabolite biosynthetic gene clusters in the genome of *S. sclerotiorum* and analysed their expression during infection of *Brassica napus* using an existing transcriptome data set. We also investigated their sequence diversity among a panel of 25 previously published *S. sclerotiorum* isolate genomes.

**Results:**

We identified 80 putative secondary metabolite clusters. Over half of the clusters contained at least three transcriptionally coregulated genes. Comparative genomics revealed clusters homologous to clusters in the closely related plant pathogen *Botrytis cinerea* for production of carotenoids, hydroxamate siderophores, DHN melanin and botcinic acid. We also identified putative phytotoxin clusters that can potentially produce the polyketide sclerin and an epipolythiodioxopiperazine. Secondary metabolite clusters were enriched in subtelomeric genomic regions, and those containing paralogues showed a particularly strong association with repeats. The positional bias we identified was borne out by intraspecific comparisons that revealed putative secondary metabolite genes suffered more presence / absence polymorphisms and exhibited a significantly higher sequence diversity than other genes.

**Conclusions:**

These data suggest that *S. sclerotiorum* produces numerous secondary metabolites during plant infection and that their gene clusters undergo enhanced rates of mutation, duplication and recombination in subtelomeric regions. The microevolutionary regimes leading to *S. sclerotiorum* secondary metabolite diversity have yet to be elucidated. Several potential phytotoxins documented in this study provide the basis for future functional analyses.

## Background

*Sclerotinia sclerotiorum* (Lib.) de Bary (Phylum Ascomycota, Class Leotiomycetes, Family Sclerotiniaceae) is a broad host range pathogen that infects over 400 plant species and causes substantial yield losses in crops worldwide. Crops affected are mainly dicotyledonous plants including oilseed rape and other brassicas, sunflower, chickpea, soybean, peanut and lentils, as well as some monocotyledonous plants such as onion and tulip [1]. Like other members of the Sclerotiniaceae, *S. sclerotiorum* spends approximately 90% of its life cycle as sclerotia: melanised hyphal aggregates that can remain viable for up to eight years in the soil and that play a major role in the disease cycle [2, 3]. Infection proceeds when sclerotia germinate either myceliogenically to directly infect a plant, or carpogenically to form an apothecium and disseminate ascospores [2]. After penetrating the plant cuticle *S. sclerotiorum* proliferates inside the host in a brief biotrophic phase (approximately 24 h in *Brassica napus* (oilseed rape)) before commencing a necrotrophic phase in which it kills plant cells, then feeds off the dead tissue [4].

The large host range of *S. sclerotiorum*, its ability to spread via wind dispersal and its persistence in the soil make this fungus a difficult pathogen to control. As a result, there is a need to better understand the molecular basis of *S. sclerotiorum* disease. One aspect of infection that has been little investigated in *S. sclerotiorum* is production of secondary metabolites: small, structurally diverse organic molecules that contribute to fungal growth and survival in diverse environments [5].

Secondary metabolites are synthesised by pathogenic fungi for defence, signalling, nutrient uptake and interfering with host cell structure and function [6]. Secondary metabolites that have been shown to contribute to the virulence of plant pathogenic fungi include siderophores, pigments and phytotoxins [7, 8]. Although it may be argued that some pigments and siderophores are primary metabolites as they are essential for survival, we refer to them as secondary metabolites in this study as a disambiguation as they are produced by genes in families frequently involved in production of secondary metabolites sensu stricto.

Siderophores are small, iron-chelating compounds used by fungi both to scavenge iron from the environment and to bind intracellular iron. Fungi require iron for many essential biochemical processes including respiration, the tricarboxylic acid cycle and the synthesis of deoxyribonucleotides, amino acids, lipids and sterols [9]. However, iron is difficult to take up due to its low solubility in aerobic, non-acidic environments, and at the same time needs careful management inside the cell due to its high reactivity in the reduced state [9]. Accordingly, both extracellular [10] and intracellular [11] siderophores have been shown to be necessary for the virulence of various plant pathogenic fungi.

The pigment melanin is important for protection of cells from environmental stressors such as ultraviolet light, and reinforcement of cell walls. In many plant pathogenic fungi, this pigment is an essential component of virulence as it allows sufficient build-up of turgor pressure in appressoria for penetration of host tissues [12].

Fungal secondary metabolite phytotoxins with a proven role in virulence include T-toxin, a linear polyketide required for *Cochliobolus heterostrophus* virulence to maize [12], and the aromatic polyketide cercosporin, a major virulence factor of *Cercospora* species that infect corn, soybeans and other plants [13]. Fungal phytotoxins known to occur in the Leotiomycetes include the sesquiterpene botrydial and the polyketide botcinic acid from *B. cinerea* (shown to have a redundant role in virulence [13]), the steroidal phytotoxin viridiol from *Hymenoscyphus fraxineus* (Helotiaceae) [14] and orthosporin, a polyketide from *Rhynchosporium orthosporum* [15].

The genes for fungal secondary metabolite biosynthesis are often clustered at one genomic locus and coregulated [16]. Biosynthetic gene clusters (BGCs) usually contain one or more key ‘backbone’ enzymes including polyketide synthases (PKSs), non-ribosomal peptide synthases (NRPSs), hybrid PKS/NRPSs, terpene synthases or dimethylallyl tryptophan synthases (DMATS), along with ‘decorating’ enzymes that modify the backbone molecule via oxidation, reduction, methylation or glycosylation. Other genes in a cluster may encode precursor biosynthesis enzymes, pathway-specific transcriptional regulators, and transporters to transport the end product out of the cell [17]. In recent years, bioinformatics tools have been developed to detect gene clusters in fungal genomes based on homology searches for the protein domains of key enzymes and accessory genes (antiSMASH [18] and SMURF [19]), gene coexpression (FunGeneClusterS [20]) and comparative genomics (MultiGeneBlast [21]).

As well as being interesting for their roles in virulence, BGCs are interesting for their roles in evolution. BGCs are frequently located near the ends of chromosomes in transposable element (TE) rich subtelomeric regions [22–24], which have high rates of recombination and mutation compared with other parts of the genome [22, 25]. Proximity of BGCs to TE rich subtelomeric regions is thought to be caused by selection for enhanced plasticity of the fungal metabolite profile in the face of a constantly changing environment [26].

The recent publication of the complete genome sequence of *S. sclerotiorum* [27] provides an opportunity to use bioinformatics tools to investigate the fungus’ secondary metabolite repertoire. The secondary metabolites that have to date been isolated from *S. sclerotiorum* are β-carotene, dihydroxynaphthalene (DHN) melanin and six aromatic phytotoxins isolated from liquid culture [28] whose roles in infection are unknown. While some genes involved in β-carotene and DHN melanin synthesis in *S. sclerotiorum* are known [29, 30], BGCs for these and the other metabolites have not been characterised.

In this study, we used existing genomic and transcriptomic data to predict and characterise BGCs in the genome of *S. sclerotiorum*. We identified 80 putative BGCs in total. Genes present in these 80 putative BGCs were enriched among those in subtelomeric regions. Subtelomeric clusters exhibited a strong association with repeat-rich genome sequence and were enriched for paralogous genes, suggesting that BGCs have evolved in recombination hotspots through duplication and neofunctionalisation. We also found that BGC genes exhibited a greater average sequence diversity and were more likely to exhibit presence / absence polymorphisms than non-BGC genes. Intriguingly, the number of secondary metabolites significantly up-regulated *in planta* relative to in vitro was much higher at later stages of infection, suggesting a significant role of secondary metabolite production in necrotrophic growth of *S. sclerotiorum*.

## Results

### The *Sclerotinia sclerotiorum* genome contains 80 putative secondary metabolite clusters

Secondary metabolite biosynthetic gene clusters are ubiquitous among fungi and may constitute an important adaptive component of the fungal genome. To determine how many secondary metabolites *S. sclerotiorum* potentially produces and aid future investigations into their functions, we used several software packages to predict secondary metabolite biosynthetic gene clusters in the *S. sclerotiorum* genome.

We found that antiSMASH predicted 87 clusters containing 1630 genes, while SMURF predicted 46 clusters containing 490 genes (Additional file 2: Table S1). Thirty SMURF clusters overlapped with antiSMASH clusters. Of the overlapping SMURF clusters, 29 contained predicted PKS, NRPS or PKS/NRPS-like backbone enzymes while one contained a DMATS, identified by both SMURF and antiSMASH. Two clusters identified by antiSMASH as fatty acid biosynthesis clusters were excluded from further analyses (Additional file 2: Table S1). These clusters contained fungal type I fatty acid synthase and type II fatty acid synthase domains, and did not contain other biosynthetic or tailoring enzymes.

The 16 SMURF clusters that were not predicted by antiSMASH, did not contain genes encoding known biosynthetic backbone enzymes and few contained tailoring enzymes, transporters or transcription factors. Therefore only the largest, 20-gene SMURF-only cluster, containing cytochrome P450, transporter and transcription factor encoding genes was included in further analyses. The other putative clusters are listed in Additional file 2: Table S1.

**Fig. 1:**
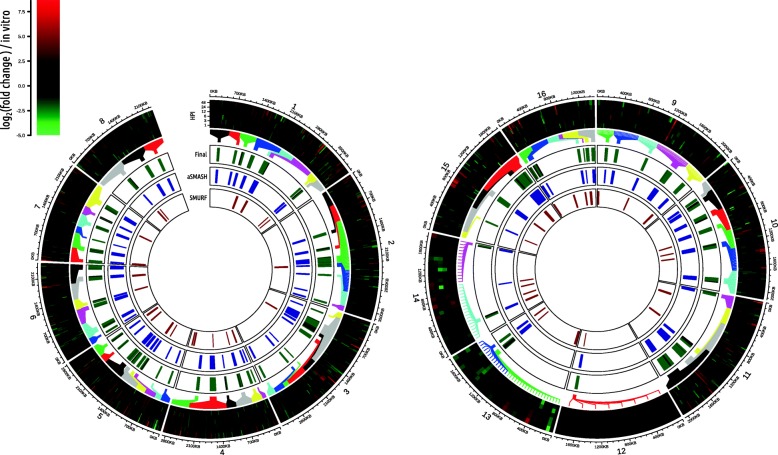
Prediction of 80 secondary metabolite biosynthesis clusters in the genome of *Sclerotinia sclerotiorum*. The left circular plot shows chromosomes 1 to 8 and the right one shows chromosomes 9 to 16. Chromosome numbers and genomic coordinates in kilobases (KB) are labelled around the peripheries of the plots. The outer-most track depicts expression data from the time course published in Seifbarghi et al. (2017) [4]. From bottom to top, the samples are 1, 3, 6, 12, 24 and 48 h post inoculation (HPI) of detached *Brassica napus* leaves. Expression data are plotted as log (fold change) relative to expression during growth in minimal medium. Log (fold change) goes from green (low) to zero (black) to red (high). The next track (‘Final’) shows the genomic coordinates of the final 80 secondary metabolite biosynthetic gene clusters (BGCs) predicted in the *Sclerotinia sclerotiorum* genome. The coloured lines emanating towards the heat map join each of the genes in the clusters to a representation of its time course expression data. The black lines represent genes that exhibited significant coexpression with their neighbours; green lines represent those that did not. The next track (‘aSMASH’), in blue, shows the positions of AntiSMASH secondary metabolite cluster predictions. The final track (‘SMURF’), in dark red, shows the positions of SMURF secondary metabolite BGC predictions. The final gene clusters depicted in track two were based on manual curation and merging of these two outputs

Secondary metabolite clusters are often transcriptionally co-regulated. Therefore, to further interrogate the antiSMASH and SMURF predictions, we also analysed expression of SM cluster genes using an existing RNA sequencing dataset profiling gene expression in *S. sclerotiorum* in vitro and during infection of *B. napus* [4]*.* We detected 174 clusters of three or more neighbouring co-regulated genes (Fig. 1, Additional file 3 Table S2), which overlapped with 37 antiSMASH-predicted clusters and 12 SMURF-predicted clusters.

To obtain a final set of putative secondary metabolite biosynthesis gene clusters based on predictions from these three software packages, we used the following procedure: 1) clusters were formed from the union of antiSMASH and SMURF predictions (with the exception of 15 SMURF-only clusters); 2) clusters were extended to include adjoining clusters of co-expressed genes; and, 3) clusters were joined if there was a gap of three or fewer genes between them. Four pairs of clusters and one set of three clusters were joined and 33 clusters extended, resulting in 80 clusters (Table 1, Fig. 1), of which 46 contained three or more co-expressed genes.

**Table 1:** The 80 secondary metabolite clusters predicted in the *Sclerotinia sclerotiorum* genome

Name	Backbone gene type	No. genes	Up-regulated *in planta*	Co-expressed	Possible product
01_1	isoprenoid	25	yes	yes	
01_2	NRPS-like protein, PKS/NRPS-like protein	14	yes	no	
01_3	PKS/NRPS-like protein	22	yes	yes	
01_4	Type I PKS (HR), NRPS-like protein	34	yes	yes	
01_5	PKS/NRPS-like protein	19	yes	no	
01_6	Type I PKS (HR), Type I PKS (HR), PKS/NRPS-like protein	29	yes	yes	
01_7	Hybrid PKS-NRPS, NRPS	12	no	no	
01_8	NRPS-like protein, PKS/NRPS-like protein	15	yes	yes	
02_1	PKS/NRPS-like protein	18	yes	no	
02_2	PKS/NRPS-like protein	20	yes	yes	
02_3	PKS/NRPS-like protein	59	yes	yes	Carotenoid
02_4	PKS/NRPS-like protein, NRPS	25	no	no	Coprogen / fusarinine (extracellular siderophore)
02_5	PKS/NRPS-like protein, Hybrid PKS-NRPS	17	yes	no	
02_6	NRPS-like protein	19	yes	no	
03_1	PKS/NRPS-like protein	9	no	yes	
03_2	isoprenoid	82	no	yes	
03_3	NRPS-like protein, PKS/NRPS-like protein	22	yes	yes	
03_4	PKS/NRPS-like protein	34	no	no	
03_5	PKS/NRPS-like protein	9	no	no	
03_6	PKS/NRPS-like protein	17	no	no	
03_7	Type I PKS (NR), PKS/NRPS-like protein	13	no	no	dihydroxynaphthalene (DHN) melanin in appressoria
04_1	PKS/NRPS-like protein	7	no	no	
04_2	Type I PKS (HR)	19	no	no	
04_3	Type III PKS, Type I PKS (HR)	28	yes	yes	
04_4	PKS/NRPS-like protein, isoprenoid	17	yes	yes	
04_5	PKS/NRPS-like protein, NRPS-like protein	65	yes	no	
04_6	isoprenoid	31	no	yes	
05_1	NRPS-like protein, PKS/NRPS-like protein	18	no	yes	
05_2	PKS/NRPS-like protein	13	no	yes	Botcinic acid
05_3	NRPS	27	no	yes	
05_4	2 x PKS/NRPS-like protein	30	yes	no	
05_5	isoprenoid	36	no	yes	
05_6	4 x PKS/NRPS-like protein	43	yes	yes	
05_7	Type I PKS (NR)	15	yes	yes	Aromatic polyketide/Sclerotinin
05_8	PKS/NRPS-like protein, Type I PKS (NR)	23	yes	yes	Aromatic polyketide/Sclerotinin
06_1	PKS/NRPS-like protein	14	yes	yes	
06_2	NRPS-like protein	32	no	yes	
06_3	2 x PKS/NRPS-like protein	33	yes	yes	
06_4	Type I PKS (HR)	21	no	yes	
06_5	other	40	no	yes	
06_6	other	39	no	yes	
07_1	Type I PKS (HR)	29	no	no	
07_2	NRPS-like protein, PKS/NRPS-like protein	28	yes	yes	
07_3	PKS/NRPS-like protein	12	no	no	
07_4	NRPS-like protein	24	yes	yes	
07_5	Type I PKS (HR), PKS/NRPS-like protein	24	yes	yes	
07_6	PKS/NRPS-like protein	44	yes	yes	
08_1	NRPS-like protein	37	no	yes	
08_2	PKS/NRPS-like protein	32	yes	no	
08_3	PKS/NRPS-like protein	25	no	no	
09_1	other	14	no	yes	
09_2	PKS/NRPS-like protein	21	yes	no	
09_3	PKS/NRPS-like protein, NRPS-like protein	17	yes	no	
09_4	PKS/NRPS-like protein	30	yes	yes	
09_5	NRPS	18	yes	no	Ferricrocin (intracellular siderophore)
10_1	PKS/NRPS-like protein	12	yes	no	
10_2	PKS/NRPS-like protein	25	yes	yes	
10_3	PKS/NRPS-like protein	26	no	yes	
10_4	PKS/NRPS-like protein	22	yes	no	
10_5	NRPS-like protein, NRPS	26	yes	yes	Epipolythiodioxopiperazine
10_6	PKS/NRPS-like protein	27	no	yes	
11_1	PKS/NRPS-like protein	16	no	no	
11_2	NRPS-like protein, PKS/NRPS-like protein	38	no	yes	
11_3	other	40	no	no	
11_4	NRPS-like protein	71	no	yes	
12_1	Type I PKS (NR)	20	yes	no	Sclerotial melanin
13_1	PKS/NRPS-like protein	20	yes	yes	
13_2	Type I PKS (HR)	14	no	no	
14_1	Hybrid PKS-NRPS (NR)	19	no	yes	
14_2	PKS/NRPS-like protein	14	no	yes	
15_1	PKS/NRPS-like protein	30	yes	no	
15_2	PKS/NRPS-like protein, Type I PKS (HR), NRPS-like protein	29	yes	no	
15_3	PKS/NRPS-like protein, Type I PKS (PR), Type I PKS (HR)	24	yes	yes	Botcinic acid
15_4	PKS/NRPS-like protein	53	no	yes	
16_1	NRPS-like protein	18	yes	yes	
16_2	isoprenoid	38	no	yes	
16_3	PKS/NRPS-like protein	15	no	no	
16_4	DMAT	5	no	no	
16_5	other	20	no	no	
16_6	PKS/NRPS-like protein	21	no	no	

Genes encoding biosynthetic backbone enzymes in the clusters included 5 NRPSs, 17 PKSs, 2 hybrid PKS-NRPSs, 96 NRPS-like and PKS/NRPS-like proteins and one DMATS (Additional file 4: Table S3a). Six clusters contained putative isoprenoid biosynthesis enzymes including three UbiA prenyltransferases, two squalene/phytoene synthases and a polyprenyl synthase. There were seven clusters with no identified backbone enzyme, while 33 clusters had two or more backbone enzymes (Additional file 4:Table S3). The majority of clusters contained either an ABC or MFS transporter (67%, *n* = 54), a Zn_2_Cys_6_ transcription factor (51%, *n* = 41), or both. Twenty-five clusters (31%) contained one or more cytochrome P450s (Additional file 4: Table S3).

### Several putative secondary metabolite biosynthesis clusters in the *Sclerotinia sclerotiorum* genome are up-regulated during infection of *Brassica napus*

Many plant pathogenic fungi produce secondary metabolites that have important roles in virulence. To assess whether this may be the case for *S. sclerotiorum*, we used a previously published transcriptome data set to determine the expression of BGCs during infection of *B. napus*. In the original analysis of the RNA sequencing dataset used here, Seifbarghi et al. [4] identified 12 PKSs, four NRPSs, five NRPS-like enzymes, a phytoene synthase and a chalcone and stilbene synthase that were up-regulated during infection of *B. napus*. All but one of these enzymes were in our predicted biosynthetic gene clusters and our analysis agrees that most are upregulated (Additional file 4: Table S3). The exceptions were three PKSs and an NRPS that were upregulated *in planta*, but not significantly, and one NRPS - here identified as an NRPS-like protein – that we found not to be upregulated.

We found that 54 backbone enzymes in 41 clusters were significantly up-regulated *in planta* at one time point or more (Fig. 1; Additional file 4: Table S3). These enzymes comprised the phytoene and chalcone/stilbene synthases identified by Seifbarghi et al. [4], 2 NRPSs, 9 PKSs, one hybrid PKS/NRPS, a UbiA prenyltransferase and 39 NRPS-like and PKS/NRPS-like proteins. Other cluster genes upregulated during infection included transcription factors (11 clusters), cytochrome P450s (16 clusters) and transporters (29 clusters). A total of 70 clusters (88%) contained at least one upregulated key gene including tailoring enzymes, transcription factors and transporters (Fig. 1). The number of upregulated backbone enzymes increased over the time course of *B. napus* infection from six at 1 h post inoculation (HPI), to 37 at 24 HPI and 33 at 48 HPI. Together these data indicate that many secondary metabolite biosynthesis clusters in *S. sclerotiorum* may have a function during plant infection, and that clusters play an increased role late in infection (> = 24 HPI).

Furthermore, analysis of the transcriptome data found 19 clusters of six or more neighbouring co-expressed genes that did not overlap with any predicted secondary metabolite clusters. This could indicate that there are potentially other biosynthesis pathways not predicted by the tools we used, that are active in *S. sclerotiorum*. However, this is quite speculative these clusters could also have other functions unrelated to secondary metabolism.

### Comparative analysis of putative secondary metabolite gene clusters provides insight into their potential functions

Numerous secondary metabolite biosynthesis genes have been predicted, and many of them functionally characterised, in many eukaryotes. To assess the homology of predicted *S. sclerotiorum* gene clusters to clusters in other eukaryotes, we conducted a MultiGeneBlast analysis. We conducted the analysis against all clusters across plant, fungal and mammalian genomes in the Genbank archive (Additional file 5: Table S4). This identified several clusters with high similarity to homologous clusters in other fungi, including clusters in the closely related fungus *B. cinerea* with known products.

Most (98 of 129; 76%) of the key biosynthetic enzymes in *S. sclerotiorum* had homologues in *B. cinerea* (54–98% amino acid identity, 51–113% query coverage per subject). This includes 7 out of 16 PKSs (77–90% amino acid identity), all 5 identified NRPSs (71 to 89% amino acid identity), a phytoene synthase and a chalcone and stilbene synthase. Four of these homologous enzymes occur in biosynthetic gene clusters that have been characterised in *B. cinerea* and that are linked to the production of melanin and the phytotoxin botcinic acid (Additional file 5 Table S4, Additional file 2: Table S1). The homologous phytoene synthase occurs in both *B. cinerea* and *S. sclerotiorum* in a four-gene putative carotenoid biosynthesis cluster. A further three homologous NRPSs have been linked to siderophore biosynthesis in *B. cinerea*, but the associated clusters have not been characterised. The following sections describe specific clusters with homology to characterised gene clusters in *B. cinerea*.

### Putative extracellular siderophore cluster

**Fig. 2:**
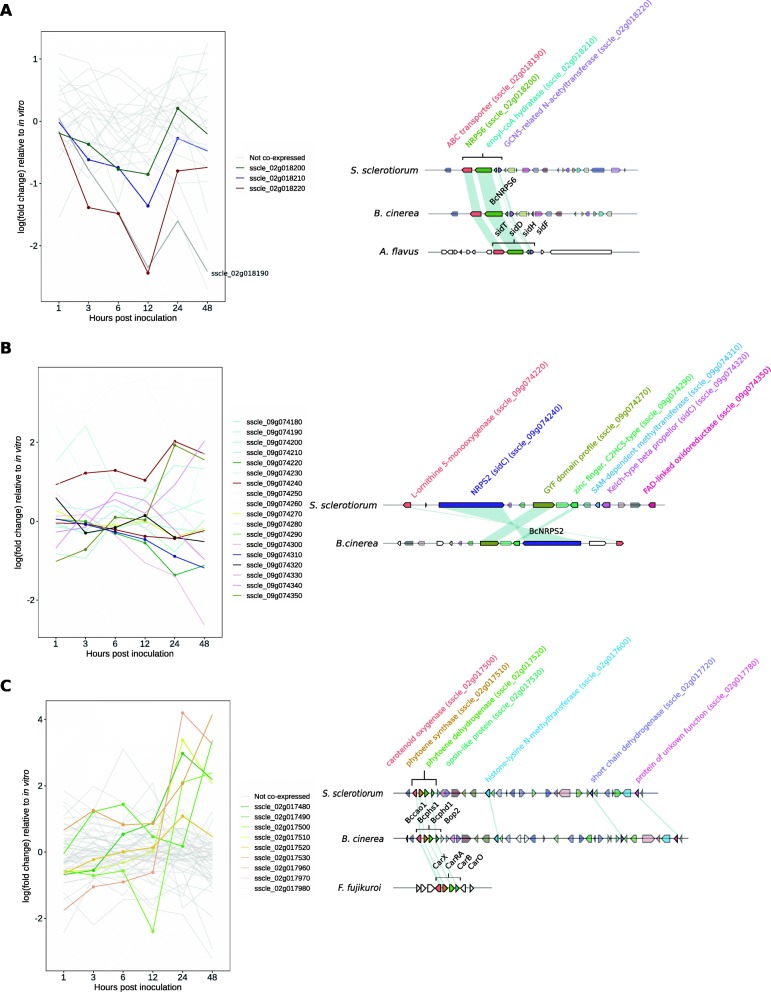
Secondary metabolite clusters with functionally characterised homologues. **a** The left plot shows log (fold change) expression relative to in vitro at six infection time points for genes in cluster 2_4. Light grey lines represent genes not significantly coexpressed. Coloured lines represent significantly coexpressed genes. The darker grey gene neighbours three coexpressed genes and has a similar expression pattern to them. The right plot illustrates cluster 2_4 gene arrangement. The top diagram represents *Sclerotinia sclerotiorum* cluster 2_4. The solid genes are conserved throughout fungi. The middle diagram is the cluster in *Botrytis cinerea*. Genes of the same colour are homologues of *S. sclerotiorum* genes. The bottom panel is the cluster in *Aspergillus nidulans*. Coloured genes represent homologues of the cluster and the white genes represent flanking genes. **b** The left plot shows the same as for A but for cluster 9_5. Lines with points are genes conserved in other fungi. Genes in this cluster were not significantly coexpressed. The right plot shows this cluster in *S. sclerotiorum* and *B. cinerea*. Non-transparent genes are in the the broadly conserved BGC, which is represented with lines and points in the expression plot to the left. Genes are coloured the same in both species if they are homologues. **c** The line graph is as per A but for genes in cluster 2_3. The grey lines represent genes in this cluster without significant coexpression. Coloured lines with points are significantly coexpressed genes. The right plot shows conservation of four genes within cluster 2_3. The top diagram is *S. sclerotiorum,* and the solid genes were significantly coexpressed. The middle diagram is *B. cinerea*. Genes of the same colour as the *S. sclerotiorum* genes are homologues. The bottom panel shows the characterised cluster in *Fusarium fujikuroi*, which contains homologues of four neighbouring genes sscle_02g017500-sscle_02g017530

We identified a putative cluster (number 2_4, Table 1, A) containing a homologue of *B. cinerea* siderophore NRPS6 and three other genes (ABC transporter, enoyl-CoA hydratase and GCN5-related N-acetyltransferase), all conserved across the Ascomycetes and known to be involved in coprogen or fusarinine biosynthesis. The *B. cinerea* gene NRPS6 has been categorised as an extracellular siderophore synthetase according to a phylogeny of NRPSs [31]. Three of the *S. sclerotiorum* genes in this cluster, sscle_02g018200 – sscle_02g018220, were significantly coexpressed according to FunGeneClusterS. These were the homologues of *B. cinerea* NRPS6 (sscle_02g018200) and two 3′ neighbouring genes. The homologue of the ABC transporter in the *B. cinerea* NRPS6 cluster (sscle_02g018190), which is the gene closest to its 5′ end, showed a similar expression pattern to these genes but was not found to be significantly coregulated (Fig. 2a). Other genes in this cluster were not coexpressed but were homologous to genes flanking the conserved extracellular siderophore cluster in *B. cinerea*.

### Putative intracellular siderophore biosynthetic gene cluster

Both NRPS2 and NRPS3 in *B. cinerea* were classified as intracellular siderophore biosynthesis NRPSs according to the phylogeny of Bushley and Turgeon [31]. We found that the homologue of the *B. cinerea* NRPS2 in *S. sclerotiorum* has a different arrangement of modules from *B. cinerea* but appears to be involved in intracellular siderophore biosynthesis since it occurs throughout the Leotiomycetes in a cluster with an l-ornithine 5-monooxygenase [32] (cluster 9_5, Table 1, Fig. 2b). Genes in cluster 9_5 that were homologous to the *B. cinerea* NRPS2 cluster showed two distinct expression patterns. The homologue of NRPS2 and an oxidoreductase were both significantly upregulated at 24–48 HPI whereas others were downregulated throughout infection with some showing an increase in expression at 48 HPI (Fig. 2b). No genes in cluster 9_5 were found to be significantly coexpressed according to FunGeneClusterS.

The putative intracellular siderophore synthase sscle_05g044190 was homologous to *B. cinerea* NRPS3, which is found in *B. cinerea* strain T4 but not in *B. cinerea* strain B05.10*.* Homologues of this NRPS and a nearby ABC transporter are clustered in some Trichocomaceae as well as in some Rutstroemiaceae and Vibrissiaceae. However, no other siderophore biosynthesis related genes were found in the cluster. This NRPS showed low expression (< 16 FPKM) and was not upregulated during *B. napus* infection.

### Putative carotenoid biosynthetic gene cluster:

Both *S. sclerotiorum* and *B. cinerea* contained a four-gene cluster with similarity to carotenoid gene clusters in *Neurospora crassa* and *F. fujikuroi* (cluster 2_3, Table 1, Fig. 2c). All four genes in this cluster were upregulated *in planta* relative to in vitro at 24 HPI and three of these genes were also upregulated at 48 HPI. These four genes and three others further downstream in cluster 2_3 were found to be significantly coexpressed with neighbouring genes but the rest of the genes in cluster 2_3 were not.

### Putative sclerotial and conidial melanin biosynthesis clusters

**Fig. 3:**
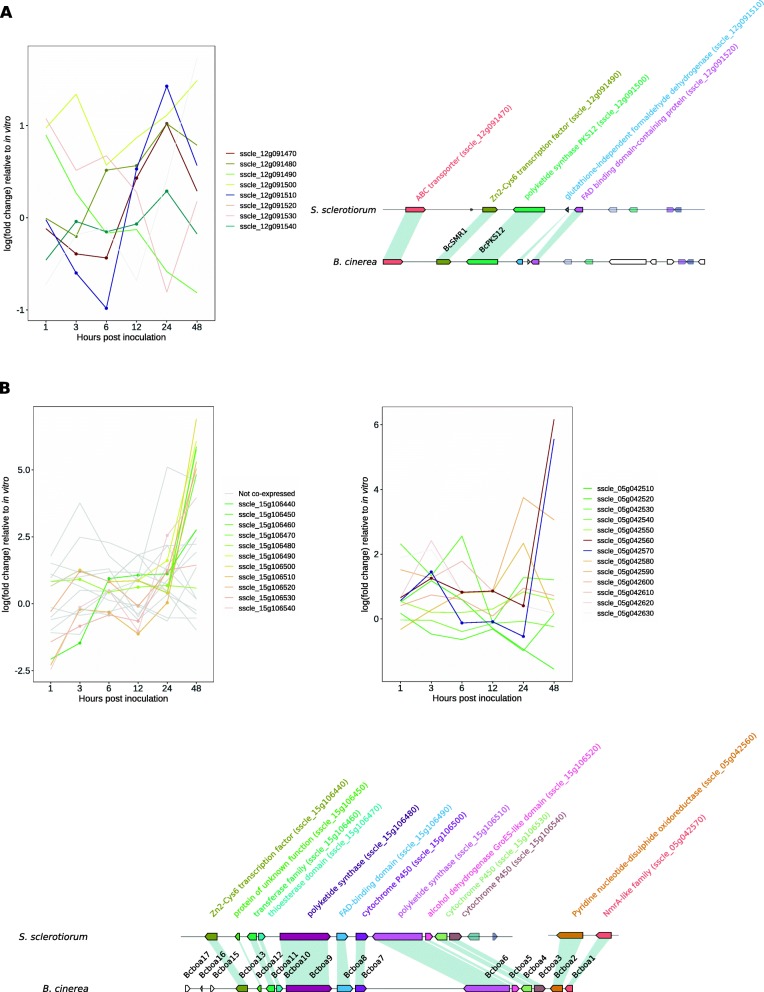
Secondary metabolite genes with functionally characterised homologues. **a** The left plot shows log (fold change) relative to in vitro at six infection time points of cluster 12_1. No genes in this cluster were significantly coexpressed. The lines with points had similar expression profiles. The right plot shows the cluster in *B. cinerea* and *S. sclerotiorum*. The solid genes are those characterised in the *B. cinerea* cluster. Genes of the same colour are homologues. **b** The left plot shows the same as A for cluster 15_3. Grey lines are non-coexpressed genes. Coloured lines with points are coexpressed genes. The right plot is the same for cluster 5_2. The lines with points are coexpressed genes in cluster 5_2. The diagram below is the cluster in *S. sclerotiorum* and *B. cinerea*. Solid genes are characterised genes in *B. cinerea*. Eleven genes in 12_1 and two genes in 15_3 were homologous to genes in the botcinic acid cluster in *B. cinerea*

PKS12 and PKS13 are homologues of *B. cinerea* dihydroxynaphthalene (DHN) melanin biosynthesis PKSs and occur in separate clusters along with homologues of other melanin biosynthesis genes identified by Schumacher [33] (Fig. 3). Cluster 12_1 contains homologues of BcPKS12 and the transcription factor BcSMR1 (sclerotial melanin regulator 1) (Table 1, Fig. 3a). BcPKS12 is hypothesised to provide the intermediate 1,3,6,8-tetrahydroxynaphthalene (T4HN) in sclerotia for conversion to DHN. Though no genes in the *S. sclerotiorum* cluster were significantly coexpressed with neighbouring genes, there was a discernible similarity between the expression profiles of sscle_12g091470 (ABC transporter) and sscle_12g091490 (Zn_2_-Cys_6_ transcription factor).

Cluster 3_7 contains homologues of BcPKS13 along with two transcription factors, a THN reductase and a scytalone dehydratase (Table 1). BcPKS13 is hypothesised to provide T4HN in conidia for conversion to DHN. This PKS showed low expression during infection (FPKM< 16).

### Botcinic acid biosynthetic gene cluster

Cluster 15_3 contains homologues of 11 of the 17 genes of the *B. cinerea* botcinic acid gene cluster (Boa3 to Boa13), while Cluster 5_2 contains another two genes (Boa1, Boa2) (Table 1, Fig. 3b). These genes were found to be coregulated despite being located on different chromosomes, with almost all genes in the cluster significantly upregulated at 48 HPI. The exception was Boa9 – one of the cluster’s two PKSs - which showed low (~ 20 FPKM) and constant expression throughout infection. Genes in these clusters outside of the homologues of the botcinic acid cluster were not significantly coexpressed according to FunGeneClusterS.

### Manual curation of domains of predicted co-regulated clusters shows that *Sclerotinia sclerotiorum* may produce ribosomally synthesised and post-translationally modified peptides

Secondary metabolites can be produced without PKSs, NRPSs and other known key biosynthetic enzymes by ribosomal synthesis, in which a precursor protein is produced ribosomally and then processed via peptidases. A number of gene clusters producing ribosomally synthesised and posttranslationally modified peptides (RIPPs) have been reported in filamentous fungi including gene clusters for the antimitotic toxins ustiloxins [34] and phomopsins [35]. Genes common to biosynthetic clusters for ustiloxins and phomopsins include copper-binding tyrosinases, zinc finger transcription-regulating proteins, S41 family peptidases, multiple DUF3328 proteins and SAM-dependent methyltransferases [35]. The ustiloxin B cluster in *A. flavus* also contains two flavin-containing monooxygenases, a cytochrome P450, an MFS multidrug transporter and a gamma-glutamyltranspeptidase.

**Fig. 4:**
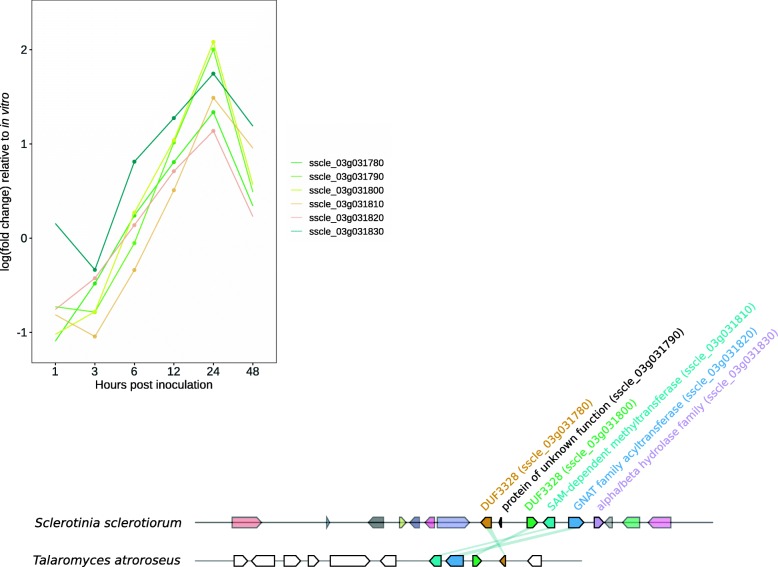
Putative ribosomally synthesised and post-translationally modified peptide. The line plot represents log (fold change) relative to in vitro for six infection time points. All genes in this group were significantly coexpressed and up-regulated at 24 and 48 h post inoculation. The plot below shows the cluster in *S. sclerotiorum* and its closest homologue, which was in *Talaromyces atroroseus*. Genes with the same colours are homologues and white ones are flanking genes in *T. atroroseus*

We conducted a preliminary investigation of whether *S. sclerotiorum* has the capacity to produce RIPPs by interrogating the Interpro annotation for proteins annotated as DUF3328, since presence of multiple DUF3328 proteins was noted as a conspicuous feature of known RIPP clusters [35]. There are four pairs of adjacent DUF3328 proteins in the *S. sclerotiorum* genome, two of which are in clusters of coexpressed genes. Genes near these pairs were then scanned for the presence of tyrosinases and peptidases. One of these clusters, which was located on chromosome 3, contained potential RIPP biosynthetic genes (Fig. 4). Eight genes in this cluster were co-expressed and significantly upregulated relative to in vitro at 24 HPI. This cluster was not conserved throughout fungi but appeared in the distantly related species *Talaromyces atroroseus*.

### *Sclerotinia sclerotiorum* secondary metabolite biosynthetic gene clusters are enriched at subtelomeres

In many species of fungi, secondary metabolites are over-represented in polymorphic and repetitive subtelomeric genomic regions [26]. This is thought to be a result of selection for enhanced metabolic plasticity in the face of a constantly changing environment. To assess whether this is the case in *S. sclerotiorum*, we assessed how many secondary metabolite cluster genes were within 300 kilobase pairs of telomeres. We found that secondary metabolite clusters were enriched in subtelomeric regions, with 38% of clusters (*n* = 30) and 29% of cluster genes being subtelomeric (chi squared test of independence χ^2^ = 23.6, degrees of freedom (df) = 1, *p* = 1.2 × 10^− 6^), compared with 24% of all genes in the genome.

**Fig. 5:**
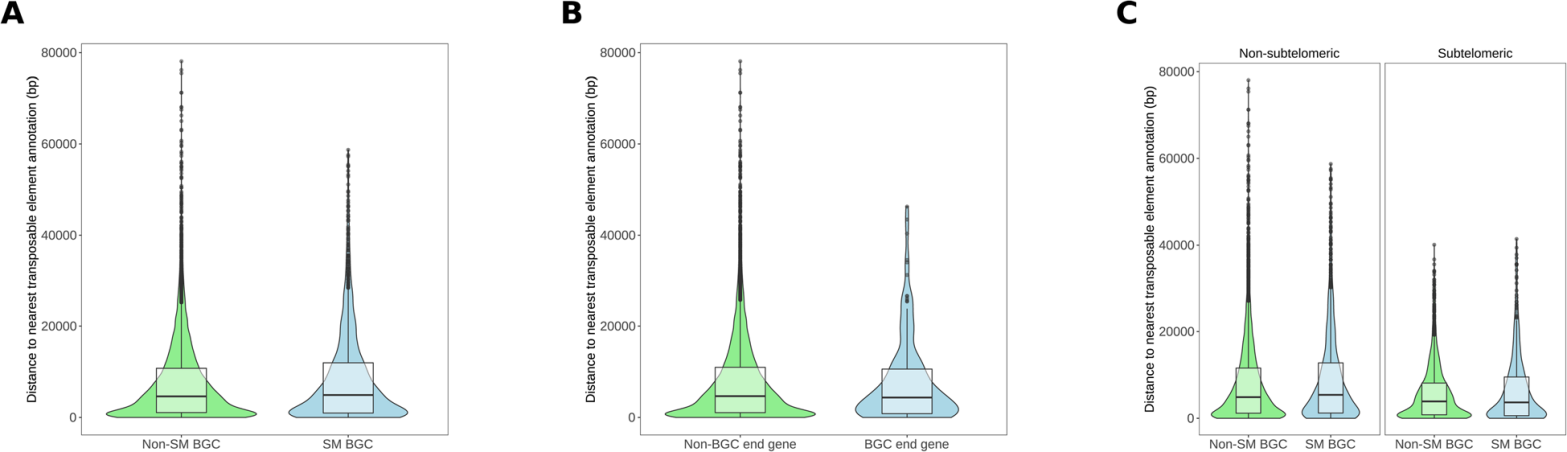
Enrichment of biosynthetic gene clusters at subtelomeres and association with repeats. **a** The y axis shows distance to nearest transposable element for non secondary metabolite BGC (Non-SM BGC) and SM BGC genes. Boxes and whiskers represent interquartile range and solid horizontal lines represent median values. The violins represent kernel density of the distribution. The points represent outlier genes (+/− 1.5 * IQR). **b** Shows the same as for A but for genes on the ends of SM BGCs. **c** Shows the same as for A and B but distinguishes between subtelomeric (fewer than 300 Kb from chromosome end) and non-subtelomeric genes

We then assessed whether secondary metabolite BGC genes were closer on average to transposable elements than non-BGC genes. We found that secondary metabolite cluster were on average further from repeats than non-cluster genes. However, when we performed the comparison using only genes on the ends of BGCs, we found that there was no difference. Regardless of whether they were in BGCs or not, genes were on average closer to transposable elements if they were within 300 Kb of telomeres (Fig. 5). The subtelomeric BGC genes were not closer to repeats than subtelomeric non-BGC genes. These data suggest that although there was a slight enrichment of BGC genes at subtelomeres, they were not especially close to transposable elements when considered as a whole gene class.

### *Sclerotinia sclerotiorum* secondary metabolite genes are more likely to be paralogues than other genes

Duplication and neofunctionalisation of genes is an evolutionary process that often affects secondary metabolites and it may occur through activity of transposable elements [26]. To determine whether *S. sclerotiorum* secondary metabolite clusters exhibited evidence of recent duplication, we detected paralogues by using OrthoFinder to find *S. sclerotiorum* genes with multiple orthologues in orthologous groups among 25 fungal genomes from 10 taxonomic classes. Of 10,336 *S. sclerotiorum* genes in orthologous groups, 3022 are paralogues, of which 687 (23%) are in secondary metabolite clusters. Chi squared tests of independence showed an association between paralogues and secondary metabolite clusters, with paralogues significantly more likely to occur in clusters than non-paralogous genes (χ^2^ = 78.1, degrees of freedom (df) = 1, *p* < 2.2e-16).

### *Sclerotinia sclerotiorum* paralogues are closer to repeats and more likely to be in taxonomically restricted orthogroups

To determine whether these paralogous genes might have been duplicated through the activity of transposable elements, we assessed their genomic positions relative to a previously published repeat annotation and subtelomeres. The mean distance of paralogues to TEs was 2278 base pairs (bp) closer than the mean distance of non-paralogues to TEs (Wilcoxon W = 15,056,993, p = p < 2.2e-16). Paralogues were also significantly more likely to be subtelomeric than non-paralogous genes (χ^2^ = 39.7, degrees of freedom (df) = 1, *p* < 3.017e-10).

As a measure of the age of duplications leading to paralogues, we assessed duplication events with respect to branches of the tree produced by the Orthofinder algorithm (Additional file 1: Figure S1); the duplication inference table from Orthofinder, ‘Duplications.tsv’, is in Additional file 6: Table S5. Overall, there were 201 duplicated genes that were specific to *S. sclerotiorum*. Of these, only 13 were not transposable element genes. Intriguingly, three of the duplicated non-transposable element genes were genes residing in BGCs. Although a relatively small number, this provides evidence of ongoing duplication of BGC genes in the *S. sclerotiorum* lineage. Since speciation between *S. sclerotiorum* and its closest relative in the tree, *S. subartcica*, duplications appeared to affect 265 genes. Of these, only 64 were not transposable element genes. A total of 16 of the non-transposable element duplicated genes were in BGCs. Although not specific to *S. sclerotiorum*, these duplication events appear to have specifically affected the *Sclerotinia* genus. Duplicated genes specific to the *Sclerotinia* genus or *S. sclerotiorum* alone were not enriched among BGCs, despite their overall enrichment among paralogous genes. This would suggest that much of the duplication and neofunctionalisation of BGCs has occurred over a relatively long evolutionary time frame with a few recent events indicative of some ongoing selection for changes in the metabolome.

### *Sclerotinia sclerotiorum* secondary metabolite biosynthetic gene clusters exhibit greater sequence diversity and presence / absence polymorphisms than other genes

**Fig. 6:**
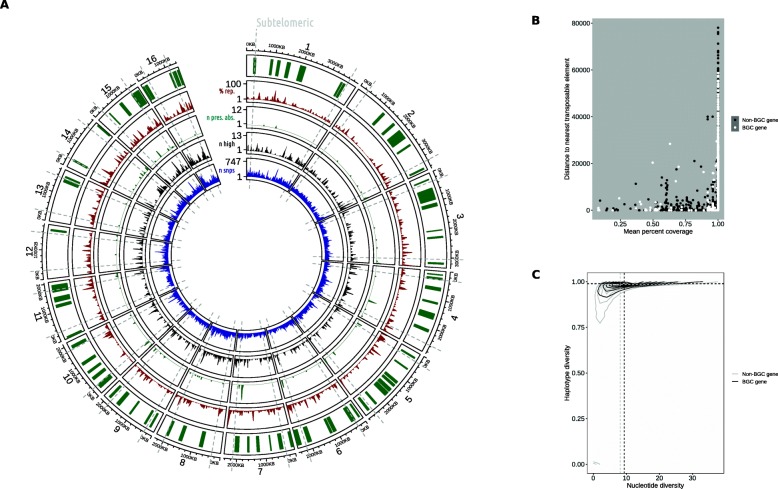
Secondary metabolite genes are more polymorphic than others. **a** Circos plot showing genomic coordinates of the 80 putative secondary metabolite biosynthetic genes (outer track in green); percentage of 50 KB windows annotated as repeat (second track in red); number of genes with presence / absence polymorphisms (third track in turquoise); number of high impact polymorphisms (fourth track in black); and, number of single nucleotide polymorphisms (SNPs) in blue. The grey dashed lines are sites fewer than 300 KB from chromosomes, defined as subtelomeric. **b** The x axis shows the average percentage of each gene that was covered by Illumina reads in a panel of 25 isolates. The y axis shows distance to nearest transposable element sequence. Non-biosynthetic gene cluster (BGC) genes are in black and BGC-genes are in white. This illustrates the enrichment of presence / absence polymorphisms among BGC genes. **c** Sequence diversity of BGC genes and non-BGC genes. The x axis shows nucleotide diversity and the y axis shows haplotype diversity. The curved lines represent kernel density of Non-BGC genes (grey) and BGC genes (black). The horizontal and vertical dashed lines represent median haplotype and nucleotide diversity, respectively, of non-BGC and BGC genes in grey and black, respectively

Since there was an enrichment of BGC genes at subtelomeres (albeit without a corresponding decrease in proximity to repeats), we hypothesised that they might be subject to accumulation of more polymorphisms than other genes. We found that secondary metabolite genes were highly over-represented among genes with presence / absence polymorphisms (*P* = 6.077e^− 5^) (Fig. 6a and b). Around 1.2% BGC genes were completely absent in at least one individual, compared with 0.4% of non-BGC genes; however, about 0.69% of BGC genes were partially absent, which was similar to the 0.77% of non-BGC genes. Despite the over-representation of BGC genes among those with complete loss in at least one isolate, there was no enrichment of BGC genes among those that exhibited at least one high impact SNP or InDel polymorphism (*P* = 0.9177) (Fig. 6a). However, the overall SNP diversity of secondary metabolite genes was higher than non-secondary metabolite genes (Fig. 6c). The mean haplotype diversity of secondary metabolite genes was 0.94, which was significantly higher than the 0.90 of other genes (*P* < 2.2e^− 16^). The mean nucleotide diversity of secondary metabolite genes was also higher at 12.3, compared with 10.91 for other genes (*P* = 4.31e^− 06^). These data indicate that *S. sclerotiorum* BGC genes are among the most polymorphic genes in the genome, affected both by point mutations and large scale insertions and deletions leading to complete gene loss.

## Discussion

Our results show some interesting potential secondary metabolite biosynthesis clusters in *S. sclerotiorum* that are transcriptionally active during infection of *Brassica napus*. Some may be linked to secondary metabolites already isolated from *S. sclerotiorum* or are homologous to clusters with known products in its close relative *B. cinerea*, while others indicate biosynthesis of yet to be identified products.

The number of PKSs, NRPSs and hybrid PKS/NRPSs found in this study is in line with those found in other plant pathogenic fungi (25, compared with 31 in *B. cinerea* and 11 to 59 in eight other plant pathogens [36–39]). Fewer studies have attempted to delineate gene clusters but our 80 clusters compare with 77 in *Col. higginsianum*, 39 to 81 in four *Aspergillus* species, 67 in *F. graminearum*, 47 in *F. fujikuroi*, 32 in *Zymoseptoria tritici* and 73 in the insect pathogen *Metarhizium anisopliae* [23, 37, 40–42].

### Clusters homologous to clusters with known functions

Carotenoids are terpenoid pigments produced by bacteria, fungi, algae and plants. In fungi, carotenoids are thought to lower oxidative stress by scavenging singlet molecular oxygen and free radicals, and they are also intermediary products in the biosynthesis of other compounds such as the chromophore retinal [43]. We found a four gene cluster occurring in both *S. sclerotiorum* and *B. cinerea* with close similarity to a characterised carotenoid and retinal biosynthesis cluster in *F. fujikuroi* [44, 45]. The cluster is highly conserved, occurring in selected orders in the Dothideomycetes and Eurotiomycetes, in all Helotiales families and in many *Fusarium* species in the Sordariomycetes. The cluster contains two genes responsible for the first steps in carotenoid biosynthesis, along with genes encoding an opsin-like protein and a carotenoid oxygenase. In *F. fujikuroi* the first two genes produce torulene as a precursor to neurosporaxanthin biosynthesis, with β-carotene – which has been isolated from *S. sclerotiorum* [46] – and γ-carotene as byproducts. The carotenoid oxygenase CarX in the cluster then synthesises retinal from torulene and β- and γ-carotene [44]. Retinal and opsin together allow fungi to respond to light, and their roles in *B. cinerea* are being investigated [45]. In *S. sclerotiorum* the carotenoid cluster genes were upregulated later in infection at 24 and 48 HPI, which may reflect the fungus responding to oxidative stress as it begins its necrotrophic phase [47] - during the transcriptome time series used here, necrotic lesions appeared on *B. napus* leaves at 24 HPI [4].

In *F. fujikuroi*, neurosporoxanthin is produced from torulene via carotenoid oxygenase CarT and an aldehyde hydrogenase CarD, both encoded by genes elsewhere in the genome [48]. Interestingly, a homologue of CarD and a gene annotated as carotenoid oxygenase occur in *S. sclerotiorum*. These two genes are coexpressed with the four-gene carotenoid cluster suggesting that neurosporoxanthin biosynthesis may be occurring in *S. sclerotiorum*.

We identified two gene clusters similar to experimentally confirmed dihydroxynaphthalene (DHN) melanin biosynthetic gene clusters in *B. cinerea* [33]. DHN melanin is a dark brown to black pigment produced by many ascomycetes that plays a role in protecting cells from radiation, heavy metals, microbial attack, cell wall degrading enzymes and reactive oxygen species [49–51]. *S. sclerotiorum* has been shown to produce DHN melanin and express scytalone dehydratase, an enzyme specific to DHN melanogenesis, both in sclerotia and in mycelia [30]. In *B. cinerea*, BcPKS12 and BcPKS13 produce the intermediate 1,3,6,8-tetrahydroxynaphthalene (T4HN) in sclerotia and conidia respectively, after which T4HN is further modified by three enzymes: two redundant THN reductases and a scytalone dehydratase [33].

There is some evidence of a similar pathway in *S. sclerotiorum*, but while *S. sclerotiorum* has homologues of the *B. cinerea* DHN melanin PKSs, their role had not been investigated until recent experiments used CRISPR-Cas9 to disrupt SsPKS13 [52]. This was the first example of a secondary metabolite backbone enzyme in *S. sclerotiorum* being knocked out. Disrupted SsPKS13 mutants produced albino compound appressoria but normal sclerotia [52], suggesting that sclerotial and mycelial melanin formation are partially independent. However when Thr1 and Scd1 (homologues of the downstream processing enzymes Bcbrn2 (a THN reductase) and Bcscd1 (scylatone dehydratase)) were disrupted, pigmentation of sclerotia was reduced but not inhibited, suggesting that Scd1 is not essential for melanin biosynthesis in *S. sclerotiorum* as it is in *B. cinerea*, and that an alternative pathway for melanin biosynthesis is available in *S. sclerotiorum*.

In the transcriptome dataset we used, the only melanogenic genes significantly upregulated were SsPKS12 and the ABC transporter in the sclerotial melanin cluster at 24 and 48 HPI. This suggests sclerotia formation began late in infection, which is in accordance with Seifbarghi et al.’s [4] observation that two sclerotia-specific proteins only detected during sclerotia formation were upregulated in *S. sclerotiorum* at 24 and 48 HPI. There was no upregulation of genes in the conidial melanin cluster responsible for melanin in compound appressoria, perhaps because melanin is not vital to plant cell wall penetration for *S. sclerotiorum*.

Siderophores are important virulence factors for pathogenic fungi, with results from a number of plant pathogens showing that mutants deficient in extracellular siderophore production are hypersensitive to oxidative stress and low iron availability [53]. Siderophores have not yet been isolated from *S. sclerotiorum*. *B. cinerea* is known to have two NRPSs – BcNRPS6 and BcNRPS2 - that group phylogenetically with extracellular and intracellular siderophore biosynthetic enzymes [31], but corresponding siderophore gene clusters have not been characterised. We found two gene clusters with NRPSs homologous to BcNRPS6 and BcNRPS2. These NRPSs are likely to produce hydroxamate siderophores - the most common class of fungal siderophores [32]. Genes with high similarity (52–82% amino acid identity, 43–100% query coverage per subject) to genes from the first gene cluster are clustered in many Eurotiomycetes and produce extracellular siderophores – either coprogen, or fusarinines [32].

The second cluster contains genes encoding an l-ornithine N^5^-monooxygenase, which is required for all hydroxamate siderophore biosynthesis, plus an NRPS characteristic of intracellular ferrichrome siderophore biosynthesis with similarity (71% amino acid identity, 100% query coverage per subject) to BcNRPS2. This NRPS may produce the ferrichrome siderophore ferrirhodin, which is the only siderophore so far isolated from *B. cinerea* [54]. Alternatively it may produce another ferrichrome family member such as ferricrocin or ferrichrome A [55].

The gene cluster for extracellular siderophore biosynthesis does not contain l-ornithine N^5^-monooxygenase, although this enzyme produces the precursors for biosynthesis. However there are three ornithine-N^5^- monooxygenase genes at other loci in the *S. sclerotiorum* genome – two in clusters 9_5 and 11_4, and one not in a cluster - that may be involved in this biosynthesis pathway. This is not unprecedented since siderophore biosynthesis genes are clustered at three different loci in *A. fumigatus* and *A. nidulans*, with ornithine-N^5^-monooxygenase at a different location to the NRPS [56].

Genes in both intracellular and extracellular siderophore clusters were downregulated *in planta* relative to in vitro, with the exception of homologues of NRPS2 in the *B. cinerea* intracellular siderophore cluster (upregulated at all time points > 1 HPI) and an FAD-linked oxidoreductase upregulated at 24 and 48 HPI. The oxidoreductase may have a role in decoupling iron from the siderophore complex so that it can be used in the cell, as observed in the bacterium *Staphylococcus aureus* [57]. Genes in the extracellular siderophore cluster reached their minimum expression levels at 12 HPI, before increasing from 12 to 24 HPI. The l-ornithine N^5^-monooxygenase and oxidoreductase genes were also steeply upregulated from 12 to 24 HPI. This may indicate that the fungus used its internal iron stores by 12 HPI before beginning metabolically costly siderophore secretion. *S. sclerotiorum* may also be repressing extracellular siderophore release during its biotrophic phase in order to avoid triggering a defence reaction in the plant, as observed in *Col. graminicola* [58]. Intracellular siderophore production may increase at a later stage to maintain iron homeostasis while extracellular siderophores are active.

### Potential phytotoxins

We found only one cluster homologous to a known toxin biosynthesis cluster: the botcinic acid cluster from *B. cinerea*. Botcinic acid is a polyketide that induces necrosis and chlorosis in plants and also has antifungal activity [13]. This cluster is upregulated at 48 HPI so it is possible that *S. sclerotiorum* is producing botcinic acid, or a related compound.

Six aromatic compounds including four aromatic polyketides have been isolated from *S. sclerotiorum* [59]. Sclerin – a phytotoxin that causes necrosis and chlorosis in susceptible species [28] - is proposed to be biosynthesised via intermediates sclerotinin A and sclerotinin B, which are closely related to citrinin [59]. Sclerone and isosclerone are naphtalenes, whose biosynthesis pathways are unknown [59]. As aromatic polyketides, sclerotinin A and B must be produced by a nonreducing Type I iterative PKS [60]. There are four nonreducing Type I PKSs encoded in the *S. sclerotiorum* genome: SsPKS12 and SsPKS13 are likely involved in melanin biosynthesis as mentioned above while the other two – SsPKS17 and SsPKS4 - are candidates for producing sclerotinin A and/or B. SsPKS17 was significantly upregulated later in infection at 12, 24 and 48 HPI, while SsPKS4 was significantly upregulated at 3, 6 and 12 HPI. Both clusters have key biosynthetic genes present throughout the Sclerotiniaceae and in isolated Trichocomaceae species. Within the Leotiomycetes, SsPKS4 in cluster 5_5 occurs only in *S. sclerotiorum* and *S. borealis*.

We identified one NRPS homologous to BcNRPS1 that belongs to the epipolythiodioxopiperazine (ETP) module 2 toxin subfamily [31]. This NRPS is upregulated at 12, 24 and 48 HPI during *B. napus* infection and occurs in a cluster homologous to a 7-gene cluster in several *Aspergillus* species. ETPs are cyclic peptides characterised by a disulphide bridge and produced by both mammal and plant pathogens [61]. Examples include gliotoxin, produced by several fungi such as *A. fumigatus*, and sirodesmin, produced by *Leptosphaeria maculans,* which causes blackleg disease in *B. napus*. Interestingly, while the toxicity of ETPs is partly due to their ability to generate reactive oxygen species through redox cycling [62], gliotoxin has been shown to alleviate oxidative stress caused by H_2_O_2_ exposure in *A. fumigatus*, which suggests these metabolites may play an important role in redox homeostasis [63]. This NRPS in *S. sclerotiorum* is therefore worth further investigation.

We noted a possible example of ribosomally synthesised and post-translationally modified peptide (RIPP) biosynthesis in the *S. sclerotiorum* genome. The first ascomycete RIPP biosynthesis cluster discovered was the ustiloxin B cluster in *A. flavus* reported in 2014 [34], although the phytotoxic ustiloxins were first isolated from rice false smut caused by the rice pathogen *Ustilaginoidea virens.* A recent study reported that gene clusters producing the RIPP class of fungal secondary metabolites appear to be widespread in filamentous fungi [35].

### Evolutionary potential of *Sclerotinia sclerotiorum* biosynthetic gene clusters

Numerous studies, including many predating the availability of genome sequence data, have indicated that fungi produce extraordinarily diverse secondary metabolites [64]. This diversity is likely the result of complex ongoing interactions with the environment. A major driver of secondary metabolite BGC diversification is the evolutionary arms race, whereby interacting organisms continually exert selection pressure on one another to develop new ways of competing, defending themselves or parasitising others [65].

Intraspecific comparisons of fungal genomes have revealed the footprints of these cycles of adaptation. For example, in the fungal species *A. fumigatus* it was found that secondary metabolite BGCs were highly divergent between 66 strains [66]. Of the 33 clusters analysed, six were found to have undergone gene content changes in at least one of the strains and 23 were found to exhibit at least one pseudogenisation event. The gene losses and pseudogenisations observed could have resulted from selective pressure in microenvironments for loss of a particular secondary metabolite. An alternate force that may lead to such outcomes is relaxation of selective pressure on secondary metabolite BGCs once they have lost their importance in the environmental niche. This would allow them to accumulate deleterious mutations without affecting the fitness of the lineage.

In *S. sclerotiorum* we found that secondary metabolites were enriched at subtelomeric loci, which are known as hotspots for recombination and genic diversity throughout Eukaryotes [67]. While secondary metabolite genes did not appear to be generally more associated with transposable elements than other genes in the genome, we found that BGCs appeared to be heterogeneous in their evolutionary history: a subset of secondary metabolite genes close to TEs was enriched in key biosynthetic genes and showed evidence of relatively recent duplication, while the majority of BGC genes were further from TEs and more conserved. The different expression profiles of these two subgroups suggest they play different roles in the lifestyle of *S. sclerotiorum* with paralogues more likely to upregulated during infection, while single-copy orthologues are constitutively expressed. In many cases both subgroups occurred within the same cluster. A possible explanation for this is the colocation of primary and secondary metabolism genes. SM production requires many cellular components – for example, coenzyme A and S-adenosylmethionine - that are produced by primary biosynthetic pathways [63] and secondary metabolism clusters in other fungi have been found to be linked via regulation and in some cases colocation. For example, the aflatoxin cluster in *Aspergillus parasiticus* is located next to and coexpressed with a sugar utilisation cluster [68] and SM biosynthesis genes in 6 *Penicillium* species were coexpressed with primary metabolic pathways producing SM precursors [69].

Secondary metabolites in general appeared to be more polymorphic than other genes. They were enriched among genes that were completely lost in at least one isolate and had overall much higher levels of sequence polymorphism than other genes. This would suggest that secondary metabolites in this species are important responders to varied and complex interactions with the environment. Recurrent rounds of selection on different metabolite profiles would lead to the observed landscape of highly variable secondary metabolite BGCs in *S. sclerotiorum*, as in several other fungi studied to date. The evolutionary drivers behind this selective pressure remain to be elucidated. As further genomic sequence data become available, future studies into the adaptive potential and genomic diversity of *S. sclerotiorum* may provide insights into the microevolutionary regimes governing extremely broad host range plant necrotrophy.

## Conclusions

Our results have highlighted a number of gene clusters with a potential role in virulence in *S. sclerotiorum* for further investigation. We also highlighted a large number of clusters that do not contain the multimodular non ribosomal peptide synthases and polyketide synthases most often associated with fungal secondary metabolites, but instead contain partial enzymes or free standing modules, most of which are upregulated *in planta* during infection of *Brassica napus*. In addition, we observed clusters of genes with no apparent backbone enzymes that are co-expressed and upregulated *in planta*. Genes in these clusters may be part of intertwined clusters that interact with PKS or NRPS clusters, or they may be engaged in other biosynthesis pathways such as those producing RIPPs. Additional transcriptome data, which was invaluable for investigating biosynthetic gene clusters in this study, may help to evince the role of these clusters. Finally, the enrichment of clusters in *S. sclerotiorum* at subtelomeric loci and the association of paralogous genes with clusters suggests that secondary metabolite clusters in this species are subject to a higher rate of change than other parts of the genome and play a role in the adaptive capacity of this species.

## Methods

### Prediction of secondary metabolite biosynthesis gene clusters in the *Sclerotinia sclerotiorum* genome

The *S. sclerotiorum* 1980 UF-70 genome was retrieved from Genbank (accession number PRJNA348385) [27]. Secondary metabolite clusters were predicted by searching for genes encoding backbone enzymes and other cluster-associated protein domains using the programs antiSMASH v.4.0 [18] with the ClusterFinder algorithm enabled, and SMURF [19]. The union of antiSMASH and SMURF clusters (103 clusters) was refined by excluding fatty acid biosynthesis clusters and clusters with no known secondary metabolism-related functional domains from further analysis. Neighbouring clusters separated by three genes or fewer were merged, resulting in 80 clusters. We decided to merge clusters that were close neighbours because investigation of cross-talk between fungal BGCs has revealed ‘superclusters’ with genes involved in multiple interconnected biosynthesis pathways, including one in *Aspergillus fumigatus* containing more than 60 genes [63, 70]. Clusters and backbone biosynthetic enzymes predicted by antiSMASH and SMURF are listed in Additional file 2: Table S1.

### Manual interrogation of gene content in *Sclerotinia sclerotiorum* secondary metabolite clusters

To identify key genes other than backbone enzymes in clusters, we compiled a list of transporters, Zn_2_Cys_6_ transcription factors, cytochrome P450 enzymes and other tailoring enzymes (oxidases, dehydrogenases, methyltransferases and acyltransferases) based on an existing Interpro annotation of *S. sclerotiorum* amino acid sequences [27]. To this list we added accessory genes identified by antiSMASH’s secondary metabolism Clusters of Orthologous Groups (smCOG) analysis that were not included in the above categories The combined list of key biosynthetic genes is given in Additional file 2: Table S1.

### Analysis of coexpression and differential expression of *Sclerotinia sclerotiorum* secondary metabolite clusters

To assess differential expression and gene cluster coexpression we used an existing RNA sequencing dataset profiling gene expression of *S. sclerotiorum* isolate 1980 in vitro and during infection of *B. napus* cultivar DH12075 at 1, 3, 6, 12, 24 and 48 h post-inoculation (HPI) (GenBank accession number GSE83935) [4]. Three biological replicates for each time point are included in this dataset. Raw paired end Illumina reads were trimmed using Trimmomatic v0.38 [71] and then mapped to the *S. sclerotiorum* genome using Hisat2 [72]. A count matrix of mapped reads per gene was produced using FeatureCounts from the Rsubread package v1.28.1 [73]. Statistical analysis of count data was performed using DESeq2 v1.18.1 [74]. Genes were considered differentially expressed where the Wald test *p* value after adjustment for multiple hypothesis testing was less than 0.05. Differential expression was considered meaningful if the fold change exceeded two.

Gene cluster coexpression was analysed using the FunGeneClusterS program [20]. The program was run for comparison using two different measures of gene expression: fragments per kilobase per million mapped reads (FPKM) for each gene, and relativised log FPKM (RL) per gene as calculated by DESeq2, with both measures averaged over the three biological samples. Altogether 9 different combinations of FPKM or RL, window size (1 to 3), number of genes skipped (1 and 3) and correlation method (Pearson or Spearman) were used and genes appearing in three or more sets of results were considered coexpressed. FunGeneClusterS results are listed in Additional file 3: Table S2.

### Homology analysis of *Sclerotinia sclerotiorum* secondary metabolite clusters

The MultiGeneBlast algorithm v1.1.14 [21] with default settings was used to detect homologous clusters in Genbank plant, fungal and mammal genome sequences and plant and fungal Whole Genome Shotgun sequences. FASTA files of proteins encoded by the *S. sclerotiorum* genome were used as input to MultiGeneBlast. Multigeneblast output is given in Additional file 4: Table S3. To compare the occurrence of clusters in different taxa, a cluster was considered to occur in a species if at least three key biosynthetic genes (according to our prepared list) including a backbone enzyme were present at the same locus, with > 50% amino acid identity and > 50% query coverage per subject compared with the corresponding *S. sclerotiorum* genes.

### Distance of secondary metabolite clusters from transposable elements and subtelomeres

To assess cluster location relative to telomeres, we defined subtelomeric clusters as clusters with genes residing within 300 kb of the chromosome end. We tested the association between secondary metabolite cluster genes and subtelomeres using a Chi squared test of independence.

To assess distance of genes to repeats we used an existing set of repeat sequences [27] identified using the REPET pipeline [75]. We first removed potential host gene sequences from the REPET output file. We used the bedtools v2.27.0 ‘merge’ tool with default settings to combine overlapping intervals of repeats into single intervals [76]. We then used the bedtools ‘closest’ tool with the ‘-d’ (report distance to nearest feature) and ‘-t first’ (report the first tie when two features have the same distance) options, to determine the distance to the nearest repeat sequence for each gene in the *S. sclerotiorum* genome.

To compare the distance to repeats of BGC genes with that of non-BGC genes, we performed a Welch’s t-test. We also performed this test for BGC end genes and non-BGC genes. We did this as we anticipated that genes far from BGC ends would be further from repeats by virtue of being clustered (provided they were in a cluster that was not disrupted by a transposable element insertion). To compare the distance to nearest repeat of subtelomeric and non-subtelomeric genes, we used analysis of variance (ANOVA) followed by a Tukey’s HSD test for pairwise differences. The factors affecting distance to repeat that we considered were 1) non-subtelomeric BGC genes, 2) subtelomeric BGC genes, 3) non-subtelomeric non-BGC genes and 4) subtelomerice non-BGC genes.

### Prediction of paralogous genes in the *Sclerotinia sclerotiorum* genome and association with secondary metabolite clusters, subtelomeres and transposable elements

Paralogous genes were predicted using OrthoFinder v2.3.3 [77] to determine orthogroups among 25 fungal genomes from 10 classes. Orthofinder results and genomes used are given in Additional file 7: Table S6. To test the association between paralogous genes and secondary metabolite clusters, and paralogous genes and subtelomeric regions of the genome, we used Chi squared tests of independence. We used Chi-square goodness of fit tests to compare the number of genes occurring in orthogroups with different numbers of classes and species.

To map gene duplication events to speciation events in the orthofinder species tree, we used the inbuilt OrthoFinder algorithm. This algorithm is a hybrid of two approaches [78, 79] that first attempts to find the most parsimonious reconciliation of subclades from the gene tree likely to contain duplications with the species tree built by STRIDE [80]. Following this, duplication events in the reconciled tree are mapped to speciation events in the STRIDE tree by considering the deepest nodes that leads to all species under the duplication node in the gene tree.

### Analysis of presence / absence polymorphisms and sequence diversity of *Sclerotinia sclerotiorum* BGC genes

To analyse genic polymorphisms between *S. sclerotiorum* strains, we used a previously published genome sequencing dataset [81]. This dataset includes genomic Illumina reads from 25 isolates of *S. sclerotiorum* sampled from geographically diverse locations around the world. These reads were mapped to the reference genome of *S. sclerotiorum* using the methods described in [81]. To detect presence / absence polymorphisms, the percentage of each gene model covered by Illumina read mappings was determined using the Python module ‘pysam’ version 0.15.0; for this analysis, we used all reads including those mapped to multiple genomic locations. Genes that were less than 50% covered by the Illumina reads were considered completely absent; those that were between 50 and 100% covered were considered partially absent and the rest were considered fully present. To determine nucleotide and haplotype diversities of genes, the R package PopGenome version 2.7.1 [82] was used. For this analysis, we used the variant call format (VCF) file derived from analyses performed in Derbyshire et al. (2019) [81]. This file contained a subset of 21 of the 25 isolates in the whole dataset that fell into the two major *S. sclerotiorum* global population clusters previously identified. Over-representation of BGC genes among those exhibiting presence / absence polymorphisms was determined using Fisher’s exact test. Welch’s t-test was used to determine differences in average sequence diversity between BGC and non-BGC genes.

## Supplementary information

**Additional file 1: Figure S1.** Age of gene duplication events in *Sclerotinia sclerotiorum*. The bar graph to the top left shows the total number of duplicated genes (y axis) originating at each node of the tree (x axis). The top panel is for all secondary metabolite biosynthesis genes whereas the bottom panel is for only secondary metabolite key biosynthetic enzymes. The tree to the right was produced using similarity between orthologous genes with the STRIDE algorithm in OrthoFinder. The nodes labelled in this tree are the nodes that appear on the x axis in the barplot.

**Additional file 2: Table S1.** SMURF and antiSMASH predicted cluster and backbone genes, and key biosynthetic genes.

**Additional file 3: Table S2.** FunGeneClusterS results.

**Additional file 4: Table S3.** BGC cluster domains, expressions profiles and homology.

**Additional file 5: Table S4.** Results from MultiGeneBlast analysis.

**Additional file 6: Table S5.** Results from OrthoFinder showing the nodes to which orthogroups correspond along with all genes in the orthogroups.

**Additional file 7: Table S6.** Proteomes used in Orthofinder analysis.

## Data Availability

The *S. sclerotiorum* 1980 UF-70 genome was retrieved from Genbank (BioProject number PRJNA348385). The RNA sequencing dataset profiling gene expression of *S. sclerotiorum* isolate 1980 in vitro and during infection of *B. napus* variety DH12075 at 1, 3, 6, 12, 24 and 48 h post-inoculation (HPI) was retrieved from GenBank (accession number GSE83935). The genome sequences of additional *S. sclerotiorum* isolates were retrieved from GenBank (BioProject numbers PRJNA516948 and PRJNA449247).

## Abbreviations

BGC: Biosynthetic gene cluster
FPKM: Fragments per kilobase per million
NRPS: Non-ribosomal peptide synthase
PKS: Polyketide synthase
SM: Secondary metabolite

## References

[1] Boland GJ, Hall R (1994). Index of plant hosts of Sclerotinia sclerotiorum. Can J Plant Pathol.

[2] Adams P. B. (1979). Ecology ofSclerotiniaSpecies. Phytopathology.

[3] Bolton MD, Thomma BPHJ, Nelson BD (2006). Sclerotinia sclerotiorum (lib.) de Bary: biology and molecular traits of a cosmopolitan pathogen. Mol Plant Pathol.

[4] Seifbarghi S, Borhan MH, Wei Y, Coutu C, Robinson SJ, Hegedus DD (2017). Changes in the Sclerotinia sclerotiorum transcriptome during infection of Brassica napus. BMC Genomics.

[5] Bignell E, Cairns TC, Throckmorton K, Nierman WC, Keller NP (2016). Secondary metabolite arsenal of an opportunistic pathogenic fungus. Philos Trans R Soc B Biol Sci.

[6] Collemare Jérôme, Lebrun Marc-Henri (2011). Fungal Secondary Metabolites: Ancient Toxins and Novel Effectors in Plant-Microbe Interactions. Effectors in Plant-Microbe Interactions.

[7] Chooi YH, Solomon PS. A chemical ecogenomics approach to understand the roles of secondary metabolites in fungal cereal pathogens. Front Microbiol. 2014;5 NOV:1–7. 10.3389/fmicb.2014.00640 PMC4237128 25477876

[8] Scharf DH, Heinekamp T, Brakhage AA (2014). Human and plant fungal pathogens: the role of secondary metabolites. PLoS Pathog.

[9] Philpott CC (1763). Iron uptake in fungi: a system for every source. Biochim Biophys Acta - Mol Cell Res.

[10] Oide S, Moeder W, Krasnoff S, Gibson D, Haas H, Yoshioka K (2006). NPS6, encoding a nonribosomal peptide synthetase involved in siderophore-mediated iron metabolism, is a conserved virulence determinant of plant pathogenic ascomycetes. Plant Cell.

[11] HOF C, EISFELD K, WELZEL K, ANTELO L, FOSTER AJ, ANKE H (2007). Ferricrocin synthesis in Magnaporthe grisea and its role in pathogenicity in rice. Mol Plant Pathol.

[12] Collemare J, Billard A, Böhnert HU, Lebrun MH (2008). Biosynthesis of secondary metabolites in the rice blast fungus Magnaporthe grisea: the role of hybrid PKS-NRPS in pathogenicity. Mycol Res.

[13] Dalmais B, Schumacher J, Moraga J, Le Pêcheur P, Tudzynski B, Collado IG (2011). The Botrytis cinerea phytotoxin botcinic acid requires two polyketide synthases for production and has a redundant role in virulence with botrydial. Mol Plant Pathol.

[14] Andersson PF, Johansson SBK, Stenlid J, Broberg A (2010). Isolation, identification and necrotic activity of viridiol from Chalara fraxinea, the fungus responsible for dieback of ash. Pathol.

[15] Penselin D, Münsterkötter M, Kirsten S, Felder M, Taudien S, Platzer M (2016). Comparative genomics to explore phylogenetic relationship, cryptic sexual potential and host specificity of Rhynchosporium species on grasses. BMC Genomics.

[16] Keller H (1997). Metabolic pathway gene clusters in filamentous Fungi. Fungal Genet Biol.

[17] Fedorova ND, Moktali V, Medema MH, Turner G (2012). Bioinformatics approaches and software for detection of secondary metabolites. Keller NP.

[18] Weber T, Blin K, Duddela S, Krug D, Kim HU, Bruccoleri R (2015). AntiSMASH 3.0-a comprehensive resource for the genome mining of biosynthetic gene clusters. Nucleic Acids Res.

[19] Khaldi N, Seifuddin FT, Turner G, Haft D, Nierman WC, Wolfe KH (2010). SMURF: genomic mapping of fungal secondary metabolite clusters. Fungal Genet Biol.

[20] Vesth TC, Brandl J, Andersen MR (2016). FunGeneClusterS: predicting fungal gene clusters from genome and transcriptome data. Synth Syst Biotechnol.

[21] Medema MH, Takano E, Breitling R (2013). Detecting sequence homology at the gene cluster level with multigeneblast. Mol Biol Evol.

[22] Palmer J, Keller N (2011). Secondary metabolism in fungi: does chromosomal location matter?. Curr Opin Microbiol.

[23] Cairns T, Meyer V (2017). In silico prediction and characterization of secondary metabolite biosynthetic gene clusters in the wheat pathogen Zymoseptoria tritici. BMC Genomics.

[24] McDonagh Andrew, Fedorova Natalie D., Crabtree Jonathan, Yu Yan, Kim Stanley, Chen Dan, Loss Omar, Cairns Timothy, Goldman Gustavo, Armstrong-James Darius, Haynes Ken, Haas Hubertus, Schrettl Markus, May Gregory, Nierman William C., Bignell Elaine (2008). Sub-Telomere Directed Gene Expression during Initiation of Invasive Aspergillosis. PLoS Pathogens.

[25] Brown CA, Murray AW, Verstrepen KJ (2010). Rapid expansion and functional divergence of Subtelomeric gene families in yeasts. Curr Biol.

[26] Raffaele S, Kamoun S (2012). Genome evolution in filamentous plant pathogens: why bigger can be better. Nat Rev Microbiol.

[27] Derbyshire M, Denton-Giles M, Hegedus D, Seifbarghy S, Rollins J, van Kan J (2017). The complete genome sequence of the Phytopathogenic fungus Sclerotinia sclerotiorum reveals insights into the genome architecture of broad host range pathogens. Genome Biol Evol.

[28] Pedras MSC, Ahiahonu PWK (2004). Phytotoxin production and phytoalexin elicitation by the phytopathogenic fungus Sclerotinia sclerotiorum. J Chem Ecol.

[29] Amselem J, Cuomo CA, van Kan JAL, Viaud M, Benito EP, Couloux A (2011). Genomic analysis of the Necrotrophic fungal pathogens Sclerotinia sclerotiorum and Botrytis cinerea. PLoS Genet.

[30] Butler MJ, Gardiner RB, Day AW (2009). Melanin synthesis by Sclerotinia sclerotiorum. Mycologia.

[31] Bushley KE, Turgeon BG (2010). Phylogenomics reveals subfamilies of fungal nonribosomal peptide synthetases and their evolutionary relationships. BMC Evol Biol.

[32] Haas H (2014). Fungal siderophore metabolism with a focus on Aspergillus fumigatus. Nat Prod Rep.

[33] Schumacher J (2016). DHN melanin biosynthesis in the plant pathogenic fungus Botrytis cinerea is based on two developmentally regulated key enzyme (PKS)-encoding genes. Mol Microbiol.

[34] Umemura M, Nagano N, Koike H, Kawano J, Ishii T, Miyamura Y (2014). Characterization of the biosynthetic gene cluster for the ribosomally synthesized cyclic peptide ustiloxin B in Aspergillus flavus. Fungal Genet Biol.

[35] Ding W, Liu W-Q, Jia Y, Li Y, van der Donk WA, Zhang Q (2016). Biosynthetic investigation of phomopsins reveals a widespread pathway for ribosomal natural products in Ascomycetes. Proc Natl Acad Sci.

[36] Islam Md, Haque Md, Islam Mohammad, Emdad Emdadul, Halim Abdul, Hossen Quazi Md, Hossain Md, Ahmed Borhan, Rahim Sifatur, Rahman Md, Alam Md, Hou Shaobin, Wan Xuehua, Saito Jennifer A, Alam Maqsudul (2012). Tools to kill: Genome of one of the most destructive plant pathogenic fungi Macrophomina phaseolina. BMC Genomics.

[37] Dallery J-F, Lapalu N, Zampounis A, Pigné S, Luyten I, Amselem J (2017). Gapless genome assembly of Colletotrichum higginsianum reveals chromosome structure and association of transposable elements with secondary metabolite gene clusters. BMC Genomics.

[38] Sbaraini N, Andreis FC, Thompson CE, Guedes RLM, Junges ângela, Campos T, et al. Genome-wide analysis of secondary metabolite gene clusters in Ophiostoma_ulmi and *Ophiostoma novo-ulmi* reveals a fujikurin-like gene cluster with a putative role in infection. Front Microbiol. 2017;8 JUN:1–12. 10.3389/fmicb.2017.01063 PMC5468452 28659888

[39] Chooi YH, Muria-Gonzalez MJ, Solomon PS (2014). A genome-wide survey of the secondary metabolite biosynthesis genes in the wheat pathogen Parastagonospora nodorum. Mycology.

[40] Janevska S, Tudzynski B (2018). Secondary metabolism in Fusarium fujikuroi: strategies to unravel the function of biosynthetic pathways. Appl Microbiol Biotechnol.

[41] Inglis D, Binkley J, Skrzypek M, Arnaud M, Cerqueira G, Shah P (2013). Comprehensive annotation of secondary metabolite biosynthetic genes and gene clusters of *Aspergillus nidulans*, *A. fumigatus*, *A. niger* and *A. oryzae*. BMC Microbiol.

[42] Sieber Christian M. K., Lee Wanseon, Wong Philip, Münsterkötter Martin, Mewes Hans-Werner, Schmeitzl Clemens, Varga Elisabeth, Berthiller Franz, Adam Gerhard, Güldener Ulrich (2014). The Fusarium graminearum Genome Reveals More Secondary Metabolite Gene Clusters and Hints of Horizontal Gene Transfer. PLoS ONE.

[43] Avalos J, Carmen LM (2015). Biological roles of fungal carotenoids. Curr Genet.

[44] Prado-Cabrero A, Scherzinger D, Avalos J, Al-Babili S (2007). Retinal biosynthesis in fungi: characterization of the carotenoid oxygenase CarX from Fusarium fujikuroi. Eukaryot Cell.

[45] Schumacher Julia (2017). How light affects the life of Botrytis. Fungal Genetics and Biology.

[46] Georgiou Christos D., Tairis Nikolaos, Polycratis Apostolos (2001). Production of β-carotene by Sclerotinia sclerotiorum and its role in sclerotium differentiation. Mycological Research.

[47] Kabbage M, Yarden O, Dickman MB (2015). Pathogenic attributes of Sclerotinia sclerotiorum: switching from a biotrophic to necrotrophic lifestyle. Plant Sci.

[48] Díaz-Sánchez V, Estrada AF, Trautmann D, Al-Babili S, Avalos J (2011). The gene carD encodes the aldehyde dehydrogenase responsible for neurosporaxanthin biosynthesis in Fusarium fujikuroi. FEBS J.

[49] Bell AA, Wheeler MH (1986). Biosynthesis and functions of fungal Melanins. Annu Rev Phytopathol.

[50] Ludwig N, Löhrer M, Hempel M, Mathea S, Schliebner I, Menzel M (2014). Melanin is not required for turgor generation but enhances Cell-Wall rigidity in Appressoria of the corn pathogen *Colletotrichum graminicola*. Mol Plant-Microbe Interact.

[51] Jahn B, Boukhallouk F, Lotz J, Langfelder K, Wanner G, Brakhage AA (2000). Interaction of human phagocytes with pigmentless Aspergillus conidia. Infect Immun.

[52] Li J, Zhang Y, Zhang Y, Yu P-L, Pan H, Rollins JA (2018). Introduction of large sequence inserts by CRISPR-Cas9 to create pathogenicity mutants in the multinucleate filamentous pathogen Sclerotinia sclerotiorum. Am Soc Microbiol.

[53] Oide Shinichi, Moeder Wolfgang, Krasnoff Stuart, Gibson Donna, Haas Hubertus, Yoshioka Keiko, Turgeon B. Gillian (2006). NPS6, Encoding a Nonribosomal Peptide Synthetase Involved in Siderophore-Mediated Iron Metabolism, Is a Conserved Virulence Determinant of Plant Pathogenic Ascomycetes. The Plant Cell.

[54] Konetschny-Rapp S, Jung G, Huschka HG, Winkelmann G (1988). Isolation and identification of the principal siderophore of the plant pathogenic fungus Botrytis cinerea. Biol Met.

[55] Haas H, Eisendle M, Turgeon BG (2008). Siderophores in fungal physiology and virulence. Annu Rev Phytopathol.

[56] Franken ACW, Lechner BE, Werner ER, Haas H, Lokman BC, Ram AFJ (2014). Genome mining and functional genomics for siderophore production in Aspergillus Niger. Brief Funct Genomics.

[57] Kobylarz MJ, Heieis GA, Loutet SA, Murphy MEP (2017). Iron uptake Oxidoreductase (IruO) uses a Flavin adenine dinucleotide Semiquinone intermediate for Iron-Siderophore reduction. ACS Chem Biol.

[58] Albarouki E, Schafferer L, Ye F, von Wirén N, Haas H, Deising HB (2014). Biotrophy-specific downregulation of siderophore biosynthesis in Colletotrichum graminicola is required for modulation of immune responses of maize. Mol Microbiol.

[59] Barber J, Garson MJ, Staunton J (1981). The biosynthesis of fungal metabolites: Sclerin, a plant growth hormone from Sclerotinia sclerotiorum. J Chem Soc Perkin Trans.

[60] Crawford J, Townsend C (2010). New insights into the formation of fungal aromatic polyketides. Nat Rev Microbiol.

[61] Gardiner DM, Cozijnsen AJ, Wilson LM, Pedras MSC, Howlett BJ (2004). The sirodesmin biosynthetic gene cluster of the plant pathogenic fungus Leptosphaeria maculans. Mol Microbiol.

[62] Brown NA, Hammond-Kosack KE, Gupta VK, Mach RL, Sreenivasaprasad S (2015). Secreted biomolecules in fungal plant pathogenesis. Fungal biomolecules: sources, applications and recent developments.

[63] Sheridan KJ, Dolan SK, Doyle S. Endogenous cross-talk of fungal metabolites. Front Microbiol. 2015;6 JAN:1–11. 10.3389/fmicb.2014.00732 PMC4283610 25601857

[64] Keller NP, Turner G, Bennett JW (2005). Fungal secondary metabolism — from biochemistry to genomics. Nat Rev Microbiol..

[65] Smanski MJ, Schlatter DC, Kinkel LL (2016). Leveraging ecological theory to guide natural product discovery. J Ind Microbiol Biotechnol.

[66] Lind AL, Wisecaver JH, Lameiras C, Wiemann P, Palmer JM, Keller NP (2017). Drivers of genetic diversity in secondary metabolic gene clusters within a fungal species. PLoS Biol.

[67] Brown CA, Murray AW, Verstrepen KJ (2010). Rapid expansion and functional divergence of Subtelomeric gene families in yeasts. Curr Biol.

[68] Yu J, Chang PK, Bhatnagar D, Cleveland TE (2000). Cloning of a sugar utilization gene cluster in Aspergillus parasiticus. Biochim Biophys Acta - Gene Struct Expr.

[69] Nielsen JC, Prigent S, Grijseels S, Workman M, Ji B, Nielsen J (2019). Comparative Transcriptome Analysis Shows Conserved Metabolic Regulation during Production of Secondary Metabolites in Filamentous Fungi. mSystems.

[70] Wiemann P, Guo C-J, Palmer JM, Sekonyela R, Wang CCC, Keller NP (2013). Prototype of an intertwined secondary-metabolite supercluster. Proc Natl Acad Sci.

[71] Bolger AM, Lohse M, Usadel B (2014). Trimmomatic: a flexible trimmer for Illumina sequence data. Bioinform.

[72] Kim D, Langmead B, Salzberg SL (2015). HISAT: a fast spliced aligner with low memory requirements. Nat Methods.

[73] Liao Y, Smyth GK, Shi W (2014). FeatureCounts: an efficient general purpose program for assigning sequence reads to genomic features. Bioinform.

[74] Love MI, Huber W, Anders S (2014). Moderated estimation of fold change and dispersion for RNA-seq data with DESeq2. Genome Biol.

[75] Quesneville H, Bergman CM, Andrieu O, Autard D, Nouaud D, Ashburner M (2005). Combined evidence annotation of transposable elements in genome sequences. PLoS Comput Biol.

[76] Quinlan AR, Hall IM (2010). BEDTools: a flexible suite of utilities for comparing genomic features. Bioinform.

[77] Emms DM, Kelly S (2015). OrthoFinder: solving fundamental biases in whole genome comparisons dramatically improves orthogroup inference accuracy. Genome Biol.

[78] Wu YC, Rasmussen MD, Bansal MS, Kellis M (2014). Most parsimonious reconciliation in the presence of gene duplication, loss, and deep coalescence using labeled coalescent trees. Genome Res.

[79] Huerta-Cepas J, Dopazo H, Dopazo J, Gabaldón T (2007). The human phylome. Genome Biol.

[80] Emms DM, Kelly S (2017). STRIDE: species tree root inference from gene duplication events. Mol Biol Evol.

[81] Derbyshire MC, Denton-Giles M, Hane JK, Chang S, Mousavi-Derazmahalleh M, Raffaele S (2019). A whole genome scan of SNP data suggests a lack of abundant hard selective sweeps in the genome of the broad host range plant pathogenic fungus Sclerotinia sclerotiorum. PLoS One.

[82] Pfeifer B, Wittelsbürger U, Ramos-Onsins SE, Lercher MJ (2014). PopGenome: an efficient Swiss army knife for population genomic analyses in R. Mol Biol Evol.

