# Supplementary figures and images for: A detailed in silico analysis of secondary metabolite biosynthesis clusters in the genome of the broad host range plant pathogenic fungus Sclerotinia sclerotiorum
Source: BMC Genomics. 2020 Jan 2;21:7. doi: 10.1186/s12864-019-6424-4 (PMC6941272; doi:10.1186/s12864-019-6424-4)

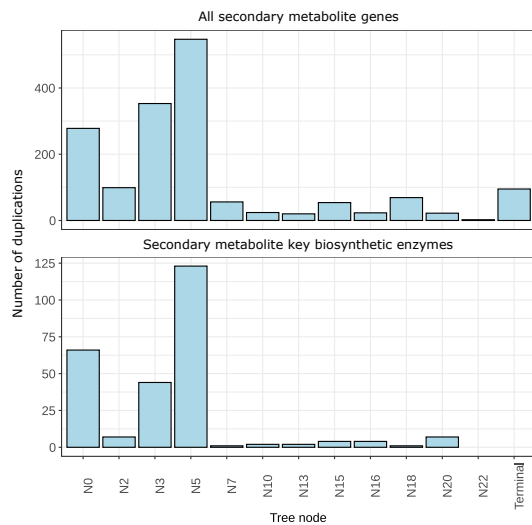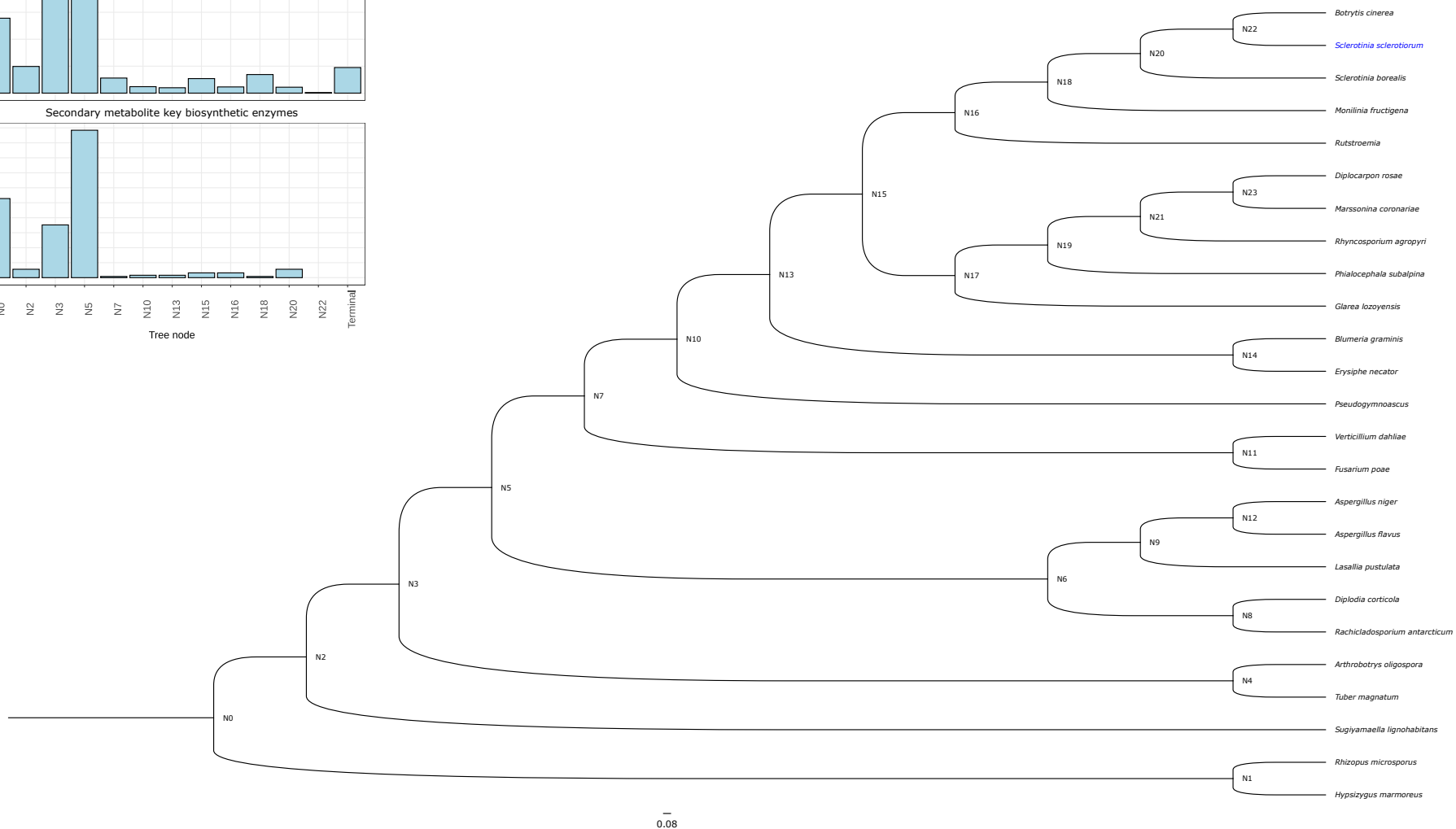

Supplement: Supplementary file 1 — Additional file 1: Figure S1. Age of gene duplication events in Sclerotinia sclerotiorum. The bar graph to the top left shows the total number of duplicated genes (y axis) originating at each node of the tree (x axis). The top panel is for all secondary metabolite biosynthesis genes whereas the bottom panel is for only secondary metabolite key biosynthetic enzymes. The tree to the right was produced using similarity between orthologous genes with the STRIDE algorithm in OrthoFinder. The nodes labelled in this tree are the nodes that appear on the x axis in the barplot. [file 12864_2019_6424_MOESM1_ESM.pdf]
